# Pancreatic Heterotopia at the Gastroesophageal Junction: A Case Report and Review of the Literature

**DOI:** 10.7759/cureus.35830

**Published:** 2023-03-06

**Authors:** Vikash Kumar, Dhir Gala, Cidney Gustke, Mili Shah, Praneeth Bandaru, Vijay Reddy Gayam, Vinaya Gadaputi, Madhavi Reddy

**Affiliations:** 1 Internal Medicine, The Brooklyn Hospital Center, Brooklyn, USA; 2 Internal Medicine, American University of the Caribbean School of Medicine, Sint Maarten, SXM; 3 Gastroenterology and Hepatology, The Brooklyn Hospital Center, Brooklyn, USA; 4 Gastroenterology and Hepatology, Blanchard Valley Health System, Findlay, USA

**Keywords:** esophagogastroduodenoscopy (egd), ectopic tissue, upper endoscopy, gastroesophageal junction, pancreatic heterotopia

## Abstract

Pancreatic heterotopia is characterized by the presence of pancreatic tissue in a location outside of its typical anatomical position. Symptoms of pancreatic heterotopia vary based on the location of the ectopic tissue. It is commonly asymptomatic and often diagnosed incidentally during routine endoscopy. Clinically significant pancreatic heterotopia is often secondary to inflammation, bleeding, obstruction, and malignant transformation. The most common location of heterotopic pancreas is within 5 cm of the pylorus usually on the greater curvature. Involvement of the gastroesophageal junction is extremely rare. In this report, we describe the case of a 57-year-old woman who was diagnosed with ectopic pancreatic tissue at the gastroesophageal junction by esophagogastroduodenoscopy after presenting with symptoms of dyspepsia.

## Introduction

Pancreatic heterotopia also referred to as ectopic, rest, accessory, or aberrant pancreas is a condition in which pancreatic tissue is not directly connected by blood supply or anatomically to the pancreas. Although it can exist at any position in the abdominal cavity, it is commonly found in the stomach, duodenum, jejunum, and gastroesophageal junction, with the greatest prevalence in the prepyloric region of the stomach. In extremely rare occurrences, cases have been reported in the mediastinum, lungs, liver, gallbladder, spleen, and esophagus [1]. The pathological origin of ectopic pancreatic tissue is still unknown but is hypothesized to arise from embryological complications because they have been reported in patients with granular pancreas, esophageal atresia, Meckel’s diverticulum, malrotation, choledochal cyst, and extrahepatic biliary atresia [2]. Typically, pancreatic heterotopia is an asymptomatic condition and is often identified incidentally on esophagogastroduodenoscopy (EGD). However, it can lead to clinical symptoms when pathological changes such as inflammation, obstruction, bleeding, and malignant transformation occur [3]. Malignancy of these lesions is incredibly rare but should be considered a differential diagnosis. A biopsy followed by histological identification is required for diagnosis, identifiable as a broad-based, well-circumscribed, submucosal tumor with normal surrounding mucosa with occasional central umbilication [4]. Overall, the presence and degree of symptoms depend on the size and location of the lesion. The most common chief complaint is epigastric abdominal pain with the potential of developing nausea, dyspepsia, heartburn, diarrhea, weight loss, hematemesis, and melena [5].

This case report was presented as an abstract at the 2023 American College of Gastroenterology Conference on 25th October 2022 (https://journals.lww.com/ajg/Fulltext/2022/10002/S2359_Pancreatic_Heterotopia_at_Gastroesophageal.2359.aspx).

## Case presentation

A 57-year-old female presented to the gastroenterology clinic for worsening esophageal reflux and epigastric pain under the rib cage. She reported worsening symptoms for the past three months. She denied nausea, vomiting, fever, chills, dysphagia, or weight loss. Her past medical history was significant for a cecal polyp, gastroesophageal reflux disease, and hyperlipidemia. She had a history of a colonoscopy two years prior, which showed external non-thrombosed hemorrhoids and a hyperplastic cecal polyp. She denied any family history of gastrointestinal or non-gastrointestinal malignancy. Social history was significant for alcohol use socially. Her current medications included atorvastatin and pantoprazole. She had no known allergies.

On presentation, her blood pressure was 123/92 mmHg, heart rate was 82 beats/minute, body mass index was 28.3 kg/m^2^, and other vitals were within normal limits. There were no significant findings on abdominal examination as it was non-tender, and she had normal bowel sounds. Her routine laboratory workup including metabolic and hematologic tests was within normal limits.

The patient was educated about lifestyle modifications and treated for dyspepsia with a proton pump inhibitor (PPI). The patient reported persistent symptoms on follow-up after six weeks and admitted to being compliant with PPI. She described occasionally feeling like food was stuck in the back of the midsternal area after swallowing. Given the concern of esophageal obstruction and worsening of symptoms, the patient was scheduled for an elective upper gastrointestinal endoscopy.

**Figure 1:**
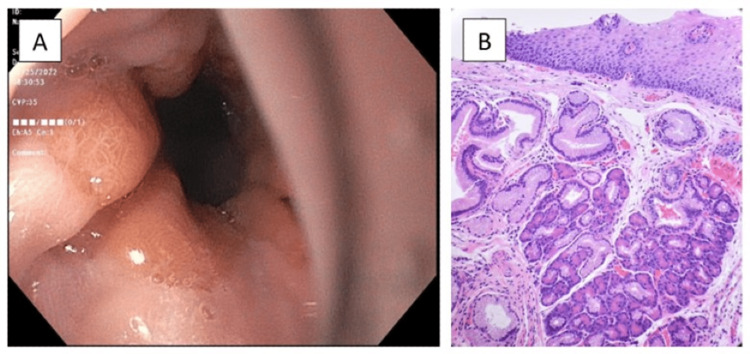
A: Esophagogastroduodenoscopy showing an irregular Z-line at the gastroesophageal junction. B: Biopsy of the gastroesophageal junction showing junctional mucosa with focal pancreatic metaplasia or heterotopia.

An EGD confirmed a diaphragmatic hernia without obstruction and reviewed normal esophagus but irregular Z-line (Figure 1A) and gastric erythema in the body and antrum. The z-line was mildly irregular and was biopsied. The histopathology of the gastroesophageal junction biopsies showed the presence of intestinal metaplasia and focal pancreatic acinar-type tissue without dysplasia (Figure 1B). Second, biopsies from the stomach revealed chronic atrophic gastritis, negative for *Helicobacter pylori*. Cold biopsies of the duodenum were also taken which were negative for *H. pylori*. The patient was managed conservatively with PPI therapy for symptomatic control and to reduce gastric inflammation. Additionally, the patient will be followed closely with surveillance measures and may require further management depending on symptoms and EGD findings.

## Discussion

Pancreatic heterotopia is defined as pancreatic tissue that lacks direct anatomical or vascular continuity with the body of the pancreas but displays a similar physiological function [2]. It has a high prevalence in middle-aged males and is normally identified within the submucosal layer of the gastrointestinal tract in the stomach, duodenum, or jejunum with rare cases at the gastroesophageal junction [5,6].

Ectopic pancreatic tissue is generally asymptomatic but may undergo characteristic pancreatic tissue complications such as acute or chronic pancreatitis, pseudocyst, and/or abscess formation causing symptoms to occur [7]. Larger lesions are more likely to cause symptoms, presenting as epigastric abdominal pain with potential nausea, dyspepsia, heartburn, diarrhea, weight loss, hematemesis, and melena [8]. In the few gastroesophageal junction cases published, patients also experienced symptoms such as gastrointestinal bleeding, dysphagia of solid food, and Boerhaave-like symptoms [9].

While the exact pathological basis of these masses remains unknown, the two most widely believed origins are explained by both the misplacement theory and metaplasia theory during embryogenesis. The misplacement theory is the idea that heterotopic pancreatic tissue develops due to displacement of tissue during foregut rotation, while the metaplasia theory theorizes that the occurrence of ectopic tissue is due to pancreatic metaplasia of the endoderm travels to the submucosa [9].

To properly diagnose pancreatic heterotopia, endoscopic ultrasound-guided fine-needle aspiration has a sensitivity between 80% and 100% and can be used to cytologically remove and evaluate the submucosal lesion [10]. In comparison, CT scans are usually non-specific and cannot provide a definitive diagnosis without a proper biopsy due its close similarity to gastric adenocarcinomas. Histopathological examination is the most important diagnostic tool to distinguish true pancreatic heterotopia from pancreatic acinar metaplasia or other differentials [9]. Biopsied pancreatic tissue can be classified into four types using the Heinrich and Gaspar-Fuentes Classification. Type I includes the presence of ducts, acini, and islets of Langerhans cells; Type II exhibits both ducts and acini; Type III exhibits ducts only; and Type IV exhibits islets only. The majority of pancreatic heterotopias are classified as Type II [11].

Although the risk of malignancy developing in heterotopic pancreas is extremely low, there have been a few cases reported in the literature. The development of pancreatic cancer has been linked to three precursor lesions, which include mucinous cystic neoplasm, intraductal papillary mucinous neoplasm, and pancreatic intraepithelial neoplasia [12]. For a carcinoma to be of pancreatic heterotopia origin, the tumor must be found within or near ectopic pancreatic tissue, the transition between pancreatic structures and carcinoma, and must contain fully developed acini and ducts [13].

The treatment options for these gastroesophageal lesions depend on their symptoms and locations, with the options of observation, Ivor-Lewis esophagectomy, and surgical or laparoscopic resection [14]. While observation has been the choice of treatment in asymptomatic cases, some studies have stated that these lesions should be removed if found incidentally during laparotomy and/or laparoscopy or if symptomatic [15,16]. Due to minimal scientific understanding of the malignancy of pancreatic ectopic tissue, a 2020 case report and literature review on heterotopic pancreas adenocarcinoma recommended excising the mass via a minimally invasive procedure to avoid future complications [15,16].

## Conclusions

This case presentation serves to highlight pancreatic heterotopia as an uncommon cause of heartburn and dyspepsia followed by food impaction. Although pancreatic heterotopia is uncommon, it must be considered a differential diagnosis in patients with heartburn and dyspepsia unresponsive to medical therapy. Treatment decisions between surgical resection or continued surveillance for monitoring of symptoms should be based on symptoms, size, and location of ectopic tissue, including any concerns for a malignant transformation. Future research should focus on the endoscopic and histological findings of pancreatic heterotopia to gain a better understanding of this condition as well as a deeper understanding of the risks of complications, such as malignant transformation. This can guide the development of more effective preventive and treatment strategies.

## References

[1] Ormarsson OT, Gudmundsdottir I, Mårvik R (2006). Diagnosis and treatment of gastric heterotopic pancreas. World J Surg.

[2] Zhang Y, Sun X, Gold JS, Sun Q, Lv Y, Li Q, Huang Q (2016). Heterotopic pancreas: a clinicopathological study of 184 cases from a single high-volume medical center in China. Hum Pathol.

[3] De Castro Barbosa JJ, Dockerty MB, Waugh JM (1946). Pancreatic heterotopia; review of the literature and report of 41 authenticated surgical cases, of which 25 were clinically significant. Surg Gynecol Obstet.

[4] Gottschalk U, Dietrich CF, Jenssen C (2018). Ectopic pancreas in the upper gastrointestinal tract: is endosonographic diagnosis reliable? Data from the German Endoscopic Ultrasound Registry and review of the literature. Endosc Ultrasound.

[5] Ogata H, Oshio T, Ishibashi H, Takano S, Yagi M (2008). Heterotopic pancreas in children: review of the literature and report of 12 cases. Pediatr Surg Int.

[6] Jenkins JK, Smith F, Mularz S, Chaudhary S (2021). Heterotopic pancreas located at the gastroesophageal junction in a hiatal hernia: a case report. Cureus.

[7] Dolan RV, ReMine WH, Dockerty MB (1974). The fate of heterotopic pancreatic tissue. A study of 212 cases. Arch Surg.

[8] Kaneda M, Yano T, Yamamoto T, Suzuki T, Fujimori K, Itoh H, Mizumoto R (1989). Ectopic pancreas in the stomach presenting as an inflammatory abdominal mass. Am J Gastroenterol.

[9] Armstrong CP, King PM, Dixon JM, Macleod IB (1981). The clinical significance of heterotopic pancreas in the gastrointestinal tract. Br J Surg.

[10] Rodriguez FJ, Abraham SC, Allen MS, Sebo TJ (2004). Fine-needle aspiration cytology findings from a case of pancreatic heterotopia at the gastroesophageal junction. Diagn Cytopathol.

[11] Thoeni RF, Gedgaudas RK (1980). Ectopic pancreas: usual and unusual features. Gastrointest Radiol.

[12] Gaspar Fuentes A, Campos Tarrech JM, Fernández Burgui JL, Castells Tejón E, Ruíz Rossello J, Gómez Pérez J, Armengol Miró J (1973). [Pancreatic ectopias]. Rev Esp Enferm Apar Dig.

[13] Maitra A, Fukushima N, Takaori K, Hruban RH (2005). Precursors to invasive pancreatic cancer. Adv Anat Pathol.

[14] Guillou L, Nordback P, Gerber C, Schneider RP (1994). Ductal adenocarcinoma arising in a heterotopic pancreas situated in a hiatal hernia. Arch Pathol Lab Med.

[15] Lowry DM, Mack TE, Partridge BJ, Barbick BC, Marks RM, Kindelan JT (2013). Thorascopic resection of esophageal heterotopic pancreas. Ann Thorac Surg.

[16] Xiong Y, Xie Y, Jin DD, Wang XY (2020). Heterotopic pancreas adenocarcinoma in the stomach: a case report and literature review. World J Clin Cases.

